# Differential Distribution of Shank and GKAP at the Postsynaptic Density

**DOI:** 10.1371/journal.pone.0118750

**Published:** 2015-03-16

**Authors:** Jung-Hwa Tao-Cheng, Yijung Yang, Thomas S. Reese, Ayse Dosemeci

**Affiliations:** 1 EM Facility, National Institute of Neurological Disorders and Stroke, National Institutes of Health, Bethesda, Maryland, United States of America; 2 Laboratory of Neurobiology, National Institute of Neurological Disorders and Stroke, National Institutes of Health, Bethesda, Maryland, United States of America; University of Iowa, UNITED STATES

## Abstract

Shank and GKAP are scaffold proteins and binding partners at the postsynaptic density (PSD). The distribution and dynamics of Shank and GKAP were studied in dissociated hippocampal cultures by pre-embedding immunogold electron microscopy. Antibodies against epitopes containing their respective mutual binding sites were used to verify the expected juxtapositioning of Shank and GKAP. If all Shank and GKAP molecules at the PSD were bound to each other, the distribution of label for the two proteins should coincide. However, labels for the mutual binding sites showed significant differences in distribution, with a narrow distribution for GKAP located close to the postsynaptic membrane, and a wider distribution for Shank extending deeper into the cytoplasm. Upon depolarization with high K^+^, neither the intensity nor distribution of label for GKAP changed, but labeling intensity for Shank at the PSD increased to ~150% of controls while the median distance of label from postsynaptic membrane increased by 7.5 nm. These results indicate a preferential recruitment of Shank to more distal parts of the PSD complex. Conversely, upon incubation in Ca^2+^-free medium containing EGTA, the labeling intensity of Shank at the PSD decreased to ~70% of controls and the median distance of label from postsynaptic membrane decreased by 9 nm, indicating a preferential loss of Shank molecules in more distal parts of the PSD complex. These observations identify two pools of Shank at the PSD complex, one relatively stable pool, closer to the postsynaptic membrane that can bind to GKAP, and another more dynamic pool at a location too far away to bind to GKAP.

## Introduction

The postsynaptic density (PSD) is a highly organized protein complex lining the postsynaptic membrane at glutamatergic synapses. A group of specialized proteins with multiple protein-protein interaction domains forms a scaffold within the PSD, around which other components can be organized [1–4]. The PSD scaffold nearest to the postsynaptic membrane consists of PSD-95 (also called SAP90) and other membrane-associated guanylate kinases (MAGUKs). Two other types of scaffold proteins, Shanks (also called ProSAP, Synamon, CortBP, Spank and SSTRIP) and Homers (also called Vesl, Cupidin, PSD-Zip45), are located deeper toward the spine cytoplasm. A group of proteins called GKAPs (also called SAPAPs) can bind both MAGUKs and Shanks, presumably pegging together the two layers of the PSD complex.

Immuno EM studies in brain localize both GKAP and Shank to the cytoplasmic side of the PSD [5–11]. Here, we focused on the interaction between GKAP and Shank in the PSD by using antibodies that recognize epitopes encompassing their mutual binding domains. We used dissociated hippocampal cultures for convenient manipulation of experimental conditions, and compared label distributions of GKAP and Shank at the PSD under different experimental conditions to assess whether Shank might lie in positions that make it unlikely to bind to GKAP.

## Materials and Methods

### Materials

**Fig 1:**
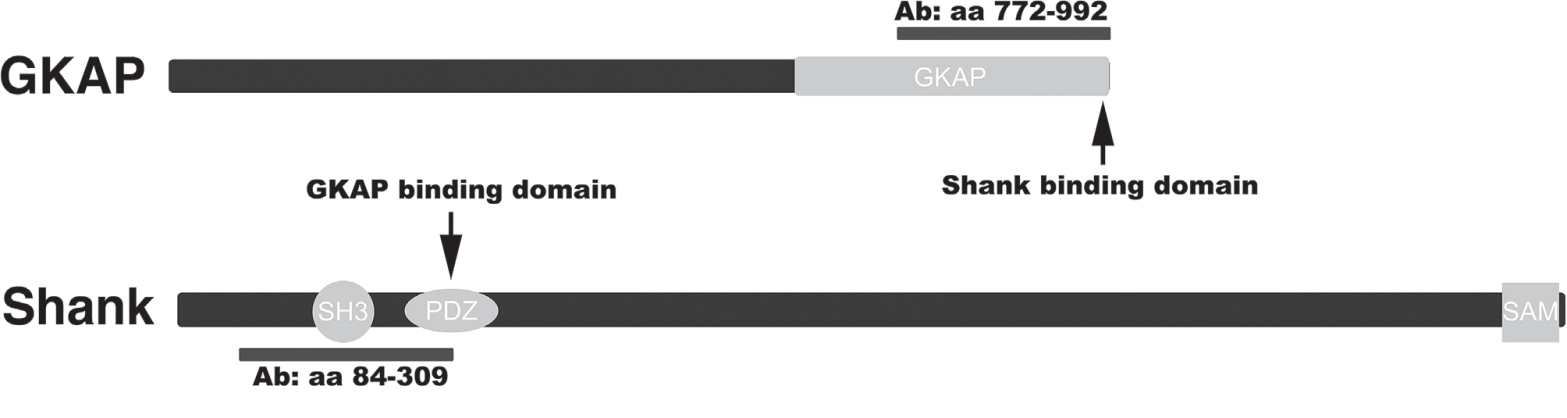
Epitopes for GKAP and Shank antibodies. The GKAP antibody used here was raised against a C-terminal peptide (aa 772–992) containing the sequence (last four residues) for its binding site to Shank. The pan Shank antibody was raised against a peptide (aa 84–309) that includes part of the sequence for its PDZ domain (aa 248–342), which is the binding site for GKAP.

Mouse monoclonal antibody against GKAP (clone N1427/31, used at 1:100), pan Shank (clone N23B/49, which recognizes all three members of the Shank family: Shank 1, 2 and 3, used at 1:250), Shank 1 (clone N22/21, used at 1:50), and Shank 2 (clone N23B/6, used at 1:200) were from NeuroMab (Davis, CA). Schematic diagram of the GKAP and Shank molecules with their mutual binding sites as well as the peptides used for the production of pan GKAP and pan Shank antibodies are illustrated in Fig. 1. The fact that peptides used for antibody production included not only their mutual binding domains, but also fairly long sequences flanking the binding domains (Fig. 1), would reduce the chances that antibody binding is blocked due to association of the two molecules.

### Dissociated hippocampal neuronal cultures and experimental conditions

The animal protocol was approved by the NIH Animal Use and Care Committee and conforms to NIH guidelines. Hippocampal cells from 21-day embryonic Sprague-Dawley rats were dissociated and grown on a feeder layer of glial cells for 3–4 weeks. During experiments, culture dishes were placed on a floating platform in a water bath maintained at 37°C. Control incubation medium was: 124 mM NaCl, 2 mM KCl, 1.24 mM KH_2_PO_4_, 1.3 mM MgCl_2_, 2.5 mM CaCl_2_, 30 mM glucose in 25 mM HEPES at pH 7.4. Wherever indicated, control medium was modified to include 90 mM KCl (compensated by reducing the concentration of NaCl) or 1 mM EGTA (calcium-free, 6.5 mM sucrose added to adjust for osmolarity). Cell cultures were washed with control medium and treated for indicated intervals with experimental media—control, high K^+^, or EGTA. Cells were fixed with 4% paraformaldehyde (EMS, Fort Washington, PA) in PBS for 30–45 min, and thoroughly washed before immunolabeling.

### Pre-embedding immunogold labeling and electron microscopy

Samples were processed as described before [12]. Briefly, fixed cells were washed with PBS, then blocked and made permeable with 5% normal goat serum and 0.1% saponin for 40–60 min, incubated with primary and secondary (at 1:200–250) antibodies (Nanogold, Nanoprobes, Yaphand, NY) for 1–2 hr, fixed with 2% glutaraldehyde in PBS for 30 min- overnight, silver enhanced (HQ kit, Nanoprobes), treated with 0.2% osmium tetroxide in 0.1M phosphate buffer at pH 7.4 for 30 min, en block stained with 0.25–0.5% uranyl acetate in acetate buffer at pH 5.0 for 1 hr, dehydrated in graded ethanols, and embedded in epoxy resin. The primary antibody was omitted in some experiments to control for nonspecific labeling by the secondary antibody.

### Sampling of synapses and morphometry

Excitatory synapses were identified by their typical structural characteristics: clustered synaptic vesicles in the presynaptic terminals, rigidly apposed synaptic cleft, and prominent postsynaptic density (PSD) [13]. At least five grid openings from each thin-sectioned sample were randomly chosen. Every cross-sectioned synaptic profile encountered was photographed with a digital CCD camera (AMT XR-100, Danvers, MA, USA). Two parameters of the distribution of label for GKAP or Shank in the PSD complex were measured: labeling intensity and distance of gold particles from the postsynaptic membrane. All measurements were statistically tested.

**Fig 2:**
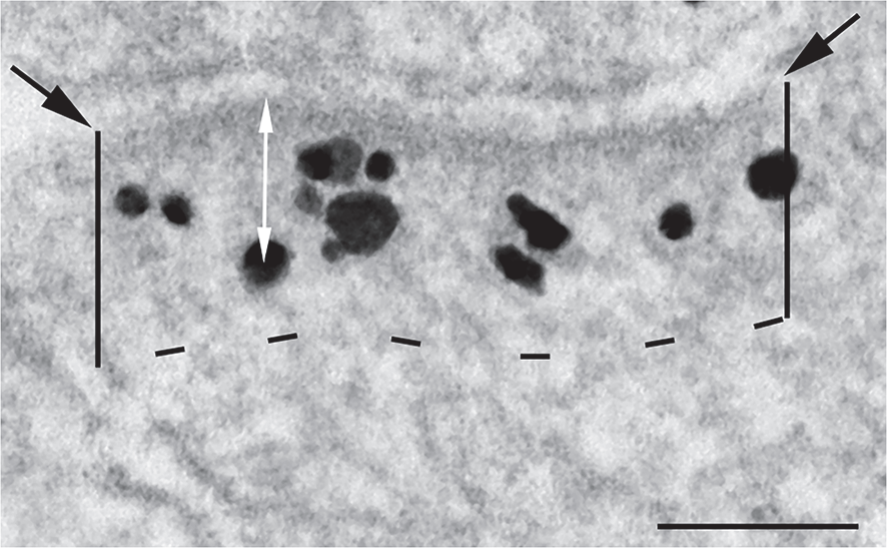
Measurements of the intensity of immunogold labeling and distance of gold particles from the postsynaptic membrane. Electron micrograph of an asymmetric synapse labeled for Shank under basal conditions. Black particles represent silver-enhanced gold particles conjugated to the secondary antibody. Areas of PSDs sampled were delineated by the postsynaptic membrane (membrane between the two black arrows), two parallel lines from the edge of the PSD extending 120 nm into the cytoplasm, and a dashed line marking the lower border at 120 nm. All labels within this area were counted and divided by the length of the PSD to yield the labeling intensity (number of label / μm PSD). Distances between gold particles and the postsynaptic membrane were measured from the center of the particle to the outer edge of the postsynaptic membrane (indicated by the white arrow). Scale bar = 100 nm.

Data on intensity of label for Shank at the PSD after depolarization with high K^+^ was from our previous study [12]. Data on intensity of label for GKAP was collected the same way as that for Shank: all particles representing label for the primary antibody located within the PSD complex were counted and divided by the length of the PSD. The area occupied by the PSD complex (referred to as zone I in our previous study [12]) was bordered by the postsynaptic membrane and enclosed by two lines perpendicular to the postsynaptic membrane, and a parallel line 120 nm from the postsynaptic membrane (Fig. 2) Mean values for intensity of label at the PSD under different conditions were compared by Student’s *t*-test (unpaired data).

Distances of gold particles from the postsynaptic membrane were measured from the center of the silver-enhanced gold particle to the outer edge of the postsynaptic membrane (white arrows in Fig. 2) for every gold particle within the PSD complex. Distance measurements were than plotted into histograms. Distance measurements for Shank were made from images collected in Tao-Cheng et al., 2010 [12]. Because the distribution of distances for Shank is skewed, comparison of the median for these non-normally distributed data was carried out with the non-parametric Wilcoxon rank-sum test (KaleidaGraph, Synergy Software, Reading, PA).

## Results

### Differences in the distributions of GKAP and Shank label under basal conditions

**Fig 3:**
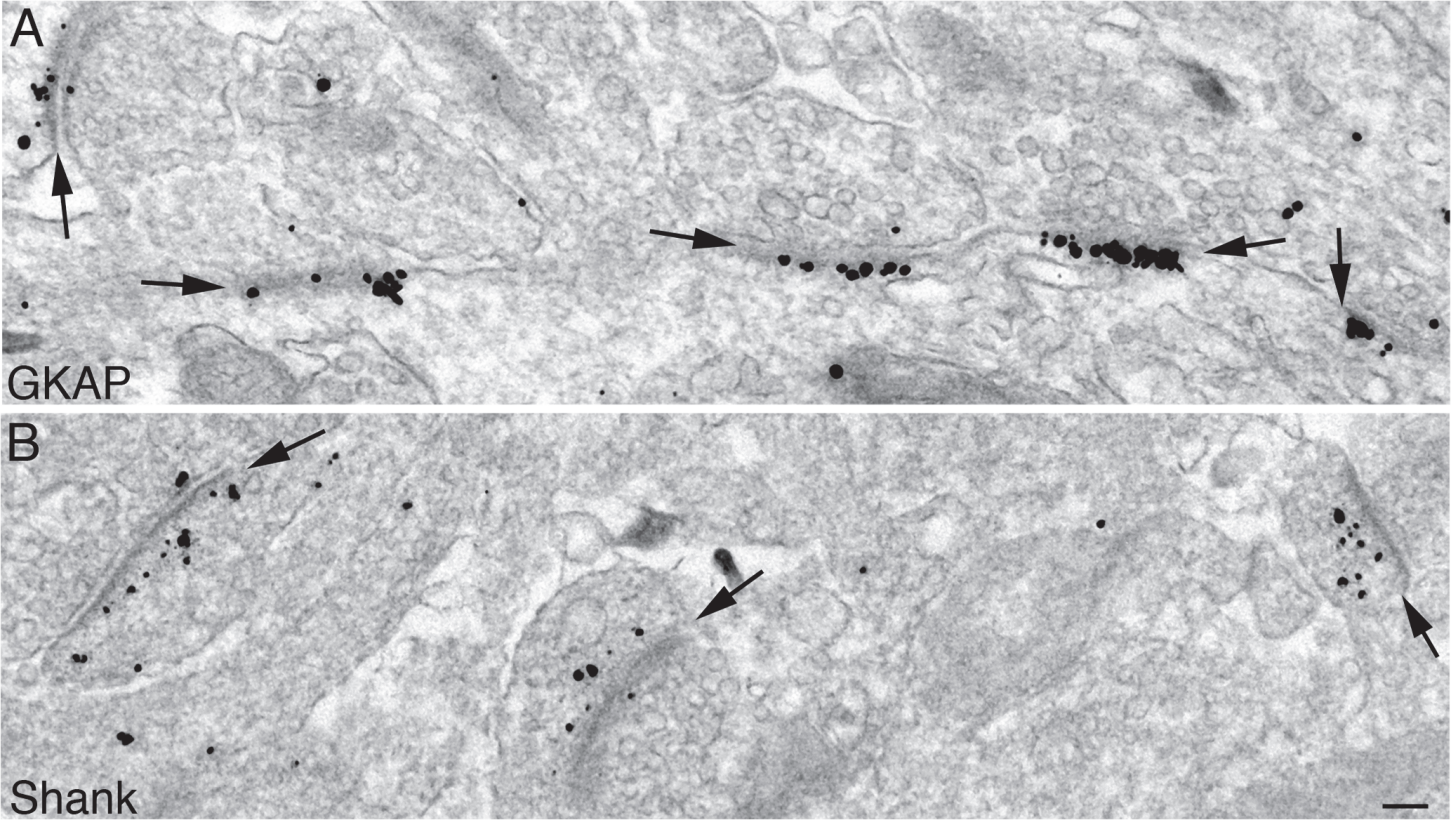
PSDs in cultured hippocampal neurons labeled for GKAP and Shank. Label for GKAP (A) is closely associated with the dense material at the core of the PSD complex (arrows), while label for Shank (B) is located in a wider area of the PSD complex (arrows) and many of the labels are distal to the dense material lining the postsynaptic membrane. Scale bar = 0.1 μm.

Pre-embedding immunogold electron microscopy was conducted on hippocampal cultures, using antibodies for GKAP and Shank. GKAP antibody was against a C-terminal peptide containing the binding site for Shank, while pan Shank antibody was raised against a peptide that includes the sequence for its PDZ domain, the binding site for GKAP (Fig. 1). PSDs of asymmetric synapses showed prominent labeling for both GKAP and Shank (Fig. 3).

**Fig 4:**
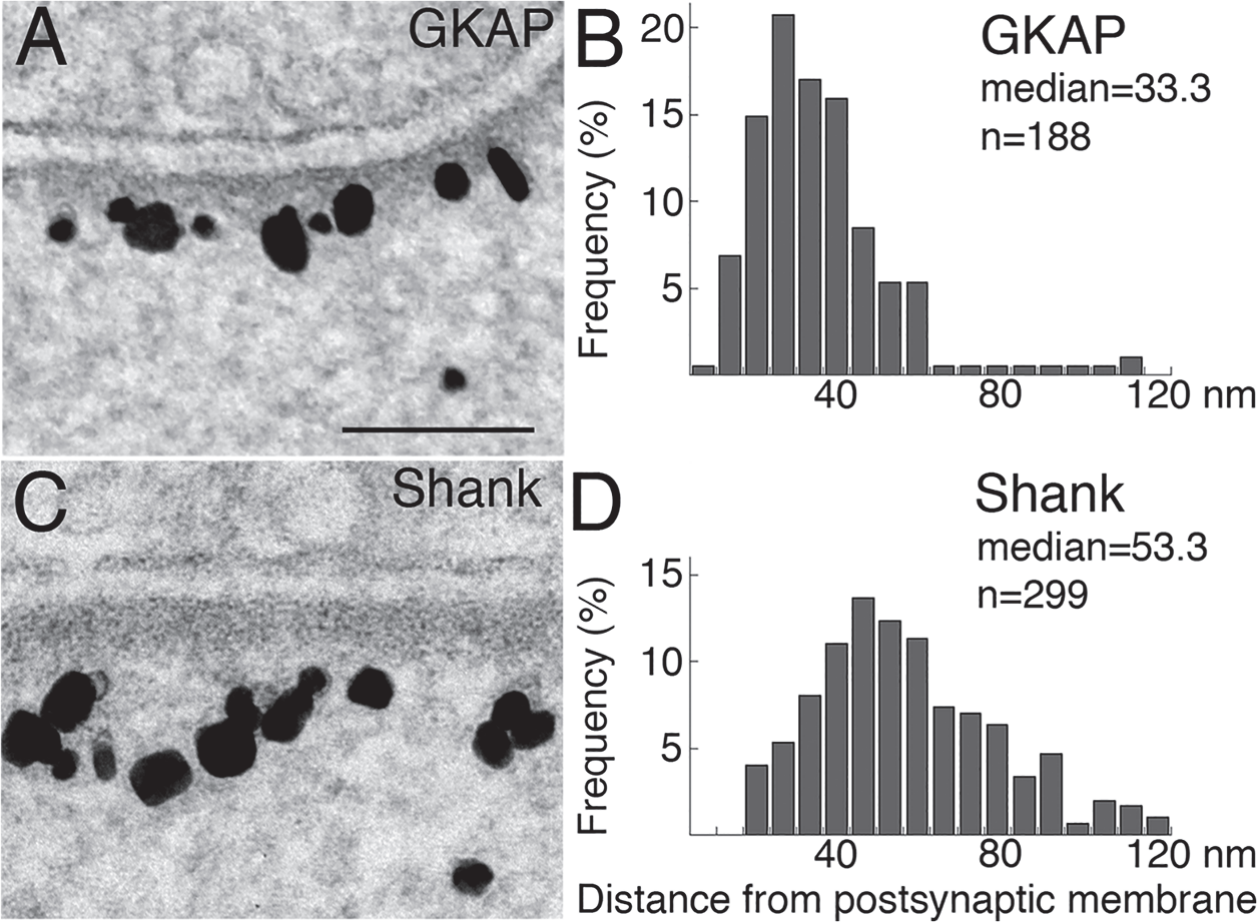
Distributions of label for GKAP and Shank are different at the PSD complex. Higher magnification EM micrographs show that under basal conditions, label for GKAP is in a narrow band closer to the postsynaptic membrane (A), while label for Shank is located in a wider band in the PSD complex (C). Scale bar = 0.1 μm. Distance measurements of label are plotted in histograms (B for GKAP and D for Shank) showing significant difference in distribution (P< 0.0001, Wilcoxon rank-sum test) with a 20 nm difference in median values.

A closer look within the PSD, however, showed significant differences in distribution, with label for GKAP located closer to the postsynaptic membrane (Fig. 4A), and label for Shank extending deeper into the cytoplasm (Fig. 4C). Under basal conditions, label for GKAP was typically within a narrower band ~ 20–50 nm from the postsynaptic membrane (Fig. 4B, median distance 33 nm, standard deviation 18 nm), while label for Shank typically appeared as a wide band ~ 30–100 nm from the postsynaptic membrane (Fig. 4D, with a median distance of 53 nm, standard deviation 23 nm).

Approximately 40% (38.6 ± 3.6%, four experiments) of label for Shank was located in a distal area of the PSD complex, 60–120 nm from the postsynaptic membrane, where very little (3.3 ± 0.7%, four experiments) label for GKAP resided. The sizable presence of Shank, as well as absence of GKAP, in the distal area of the PSD complex suggests that Shank molecules in this distal area may not be bound to GKAP.

### Distribution of GKAP does not change at the PSD after depolarization with high K^+^

**Fig 5:**
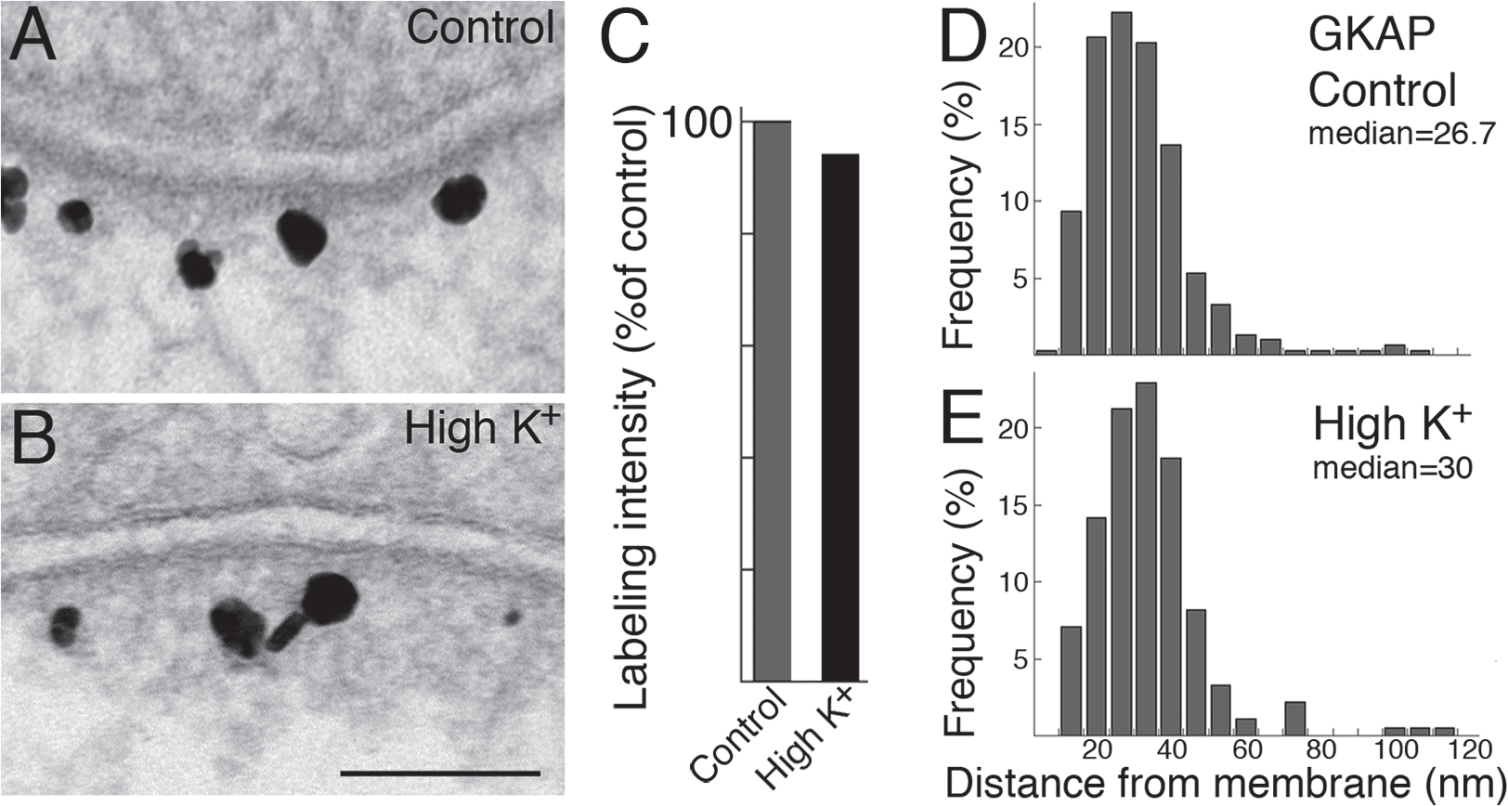
Distribution of label for GKAP is unchanged upon depolarization with high K^+^. Label for GKAP showed similar intensity under control (A) and high K^+^ (B, 90 mM K^+^, 2 min) conditions, and label remained located within a narrow band ~20–50 nm from the postsynaptic membrane. Scale bar = 0.1 μm. Labeling intensity in the PSD complex was normalized to control values and graphed in C showing no significant difference after depolarization with high K^+^. Distance measurements of label were plotted in histograms. The distributions of label for GKAP were similar under control (D) or high K^+^ (E) conditions.

The distribution of label for GKAP typically remained unchanged upon depolarization with high K^+^ (90 mM, 2 min; Fig. 5B vs. A). There was very little change in labeling intensity of GKAP at the PSD (Fig. 5C; 94 ± 7% of control values, four experiments; Table 1). Distance measurements of GKAP labels from the postsynaptic membrane yielded similar histograms under control (Fig. 5D) and high K^+^ (Fig. 5E) conditions, and the median distance of gold particles from the postsynaptic membrane remained essentially unchanged (Table 1). As with the control samples, most labels for GKAP remained within 60 nm of the postsynaptic membrane after depolarization with high K^+^ (96.7 ± 1.3% and 96.0 ± 0.6% of label in control and high K^+^ conditions, respectively, four experiments).

**Table 1:** Labeling intensity and median distance of label for GKAP in the PSD complex under control and high K^+^ conditions.

	Labeling intensity (# label/μm PSD) mean ± SEM (n) = number of synaptic profiles measured		Median distance (nm) from postsynaptic membrane (n) = number of labels measured	
	control	High K^+^	Control	High K^+^
**Exp 1**	23.4 ± 1.6 (58)	21.6 ± 1.6 (44) N.S.	26.7 (300)	30.0 (183) P<0.05
**Exp 2**	19.7 ± 2.0 (30)	20.8 ± 1.3 (47) N.S.	33.3 (188)	26.7 (295) N.S.
**Exp 3**	23.6 ± 2.3 (30)	17.7 ± 1.6 (46) P<0.05	26.7 (140)	33.3 (215) N.S.
**Exp 4**	17.5 ± 1.5 (44)	17.8 ± 2.3 (33) N.S.	30.0 (191)	30.0 (150) N.S.
**mean**	**21.0 ± 1.5**	**19.5 ± 1.0**	**29.2 ± 1.6**	**30 ± 1.3**

Statistical analysis: labeling intensity, Student’s t-test; median distance, Wilcoxon rank-sum test. N. S.: not significant.

### Shank is preferentially added to the distal area of the PSD complex after depolarization with high K^+^

**Fig 6:**
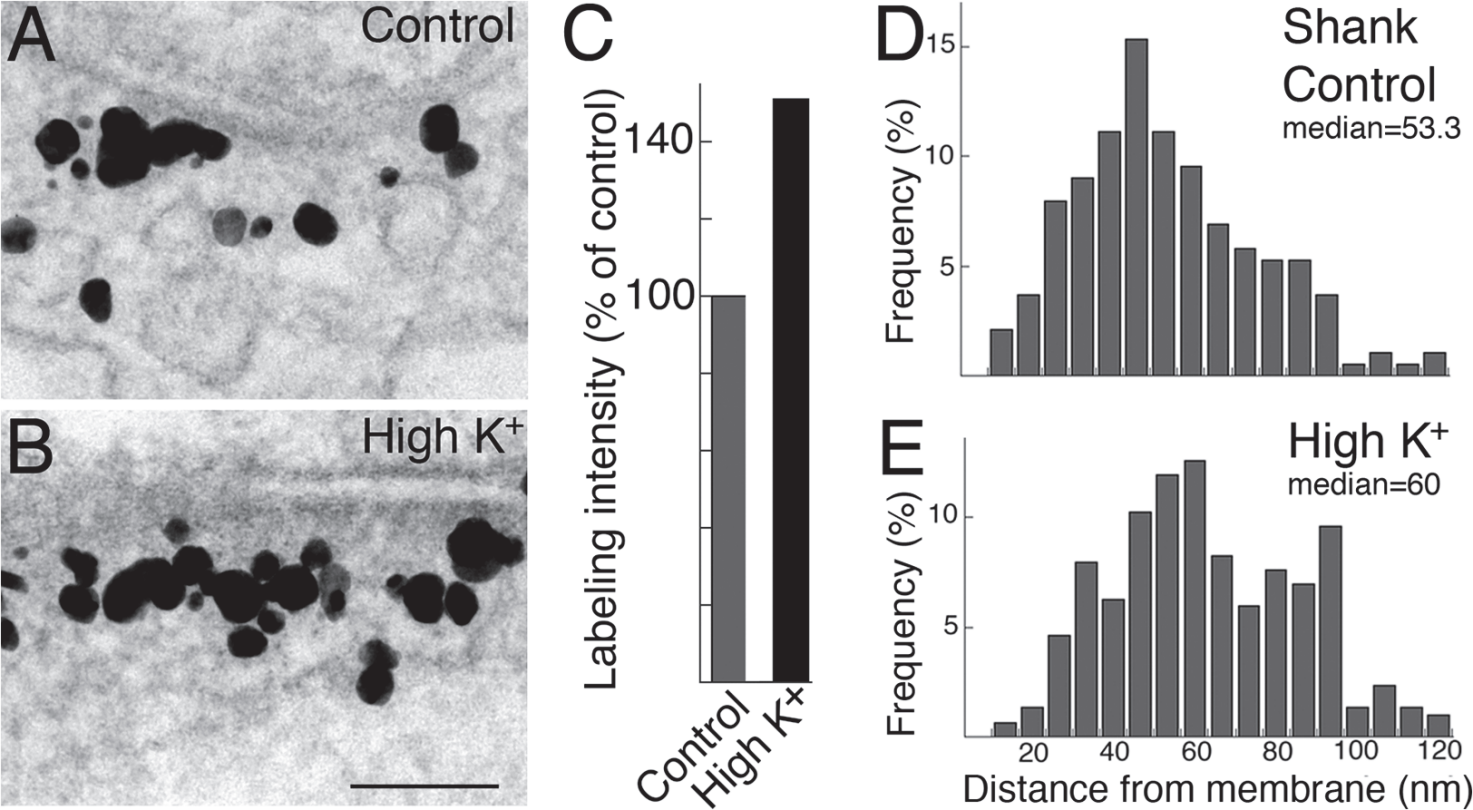
Label for Shank increased in the distal area of the PSD complex after depolarization with high K^+^. Upon depolarization with high K^+^, labeling intensity with the pan Shank antibody at the PSD increased (B vs. A, Scale bar = 0.1 μm.) to ~150% of control values (C, data from [12]; three experiments, P<0.001, paired t-test). Histograms of distance measurement of label for Shank showed a shift to the right (E vs. D) with an increase in the median distance after high K^+^ (P<0.005, Wilcoxon rank-sum test).

As reported previously [12] labeling intensity of Shank at the PSD complex increases significantly after depolarization with high K^+^ (Fig. 6A-C). Here, we measured the distances of Shank labels from the postsynaptic membrane under control and high K^+^ conditions. The histograms (Fig. 6D & E, from one experiment) show a significant increase in median distance after high K^+^ treatment, and this increase is consistent in three experiments (Table 2). Thus, depolarization preferentially promotes addition of Shank molecules into the distal area of the PSD complex, where few GKAP molecules are present (cf. Fig. 5E).

**Table 2:** Median distances of label for Shank (pan Shank antibody) in the PSD complex after depolarization with high K^+^.

	Median distance (nm) from postsynaptic membrane (n = number of labels measured)	
	**Control**	**High K** ^**+**^
**Exp 1**	57.8 (377)	63.7 (279) P<0.005
**Exp 2**	53.3 (283)	60.0 (350) P<0.005
**Exp 3**	50.0 (189)	60.0 (302) P<0.0001
**mean**	**53.7 ± 2.3**	**61.2 ± 1.2**

Statistical analysis: Wilcoxon rank-sum test.

### Shank is preferentially depleted from the distal area of the PSD complex in low calcium conditions

**Fig 7:**
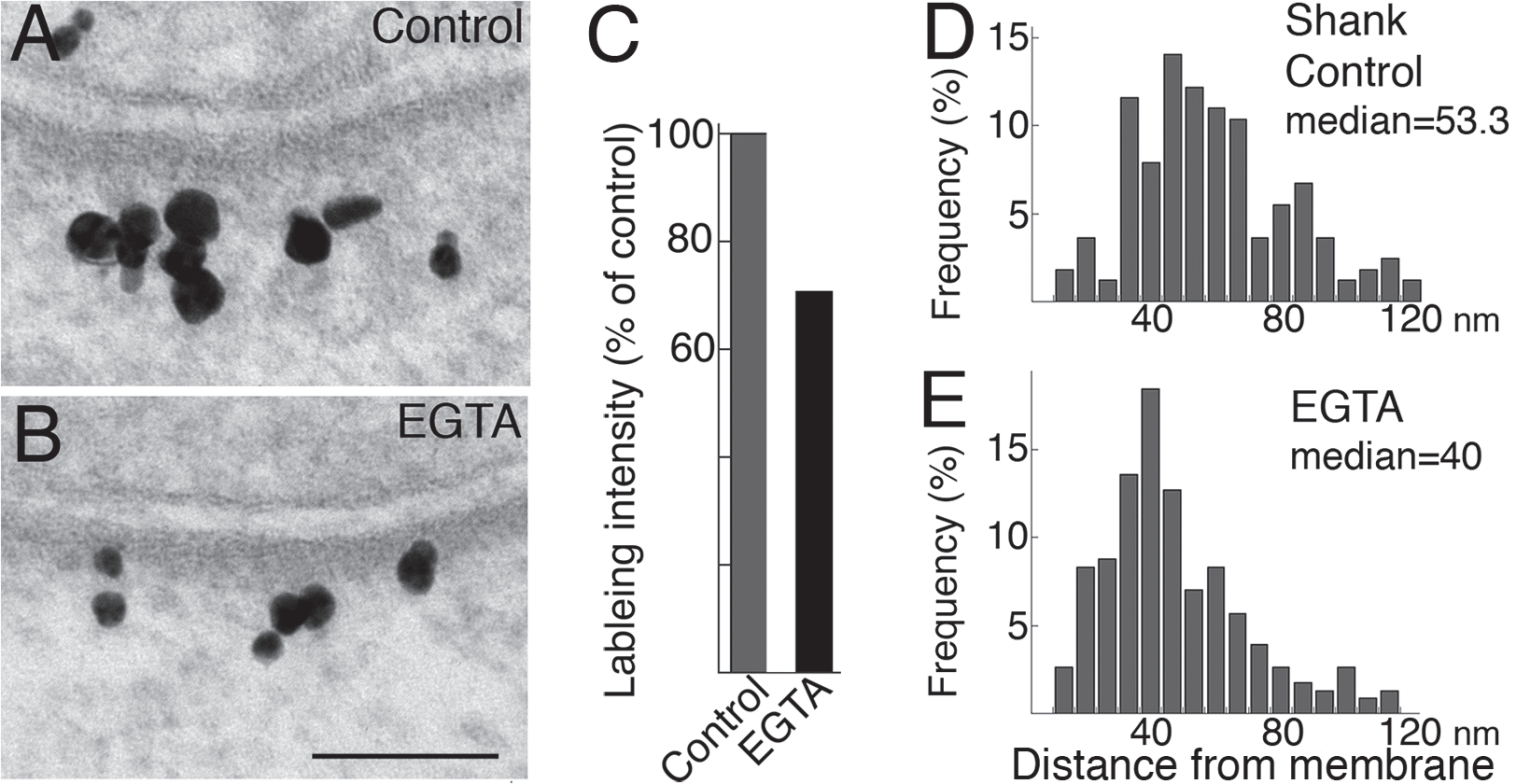
Label for Shank decreased in the distal area of the PSD complex after EGTA treatment. After EGTA treatment (5 min), labeling intensity with the pan shank antibody at the PSD decreased (B vs. A, scale bar = 0.1 μm) to ~70% of control values (C, four experiments, P<0.01). Histograms of distance measurements showed a shift to the left with a decrease in median distance after EGTA treatment (E vs. D, P<0.0001, Wilcoxon rank-sum test).

Depolarization-induced accumulation of Shank at the PSD is calcium dependent [12]. We here examine whether Shank redistributes in low calcium conditions. In samples treated with EGTA (5 min), labeling intensity of Shank at the PSD decreased (Fig. 7B) compared to control samples (Fig. 7A).

The decrease in labeling intensity for Shank at the PSD in EGTA-treated samples (71.1 ± 4.9% of control, Fig. 7C) was statistically significant in all four experiments (Table 3). Histograms of distances of Shank label from postsynaptic membrane indicate a change after EGTA treatment (Fig. 7E vs. D). The decrease in median distance was statistically significant in three out of four experiments (Table 3). These results indicate that there is a preferential loss of Shank molecules in the distal area of the PSD complex after EGTA treatment.

**Table 3:** Labeling intensity and median distance of label for Shank (pan Shank antibody) in the PSD complex under control and EGTA conditions.

	Labeling intensity (# label/μm PSD) Mean ± SEM (n = number of synaptic profiles measured)		Median distance (nm) from postsynaptic membrane (n = number of labels measured)	
	Control	EGTA	Control	EGTA
**Exp 1**	17.9 ± 1.4 (23)	12.0 ± 1.5 (17) P<0.01	57.8 (377)	52.9 (334) P<0.05
**Exp 2**	26.6 ± 2.1 (27)	19.8 ± 1.4 (48) P<0.01	53.3 (164)	40.0 (228) P<0.0001
**Exp 3**	26.9 ± 1.5 (47)	22.3 ± 1.3 (62) P<0.05	46.7 (227)	45.0 (343) N.S.
**Exp 4**	24.7 ± 1.7 (65)	14.7 ± 1.3 (51) P<0.0001	53.3 (311)	36.7 (144) P<0.0001
**mean**	**24.0 ± 2.1**	**17.3 ± 2.3**	**52.8 ± 2.3**	**43.7 ± 3.5**

Statistical analysis: labeling intensity, Student’s t-test; median distance, Wilcoxon rank-sum test. N. S.: not significant.

### Redistribution of Shank 1 and Shank 2 after high K^+^ or EGTA treatment

Our observations using a pan Shank antibody that recognizes all three isoforms (Shank 1, 2, 3) imply the existence of two pools of Shank at the PSD complex with the distal pool of Shank preferentially affected by the experimental manipulations. Since Shank 1 and 2 have different properties [14–16], we tested whether these two isoforms of Shanks are differentially located in the two pools in the PSD complex.

**Fig 8:**
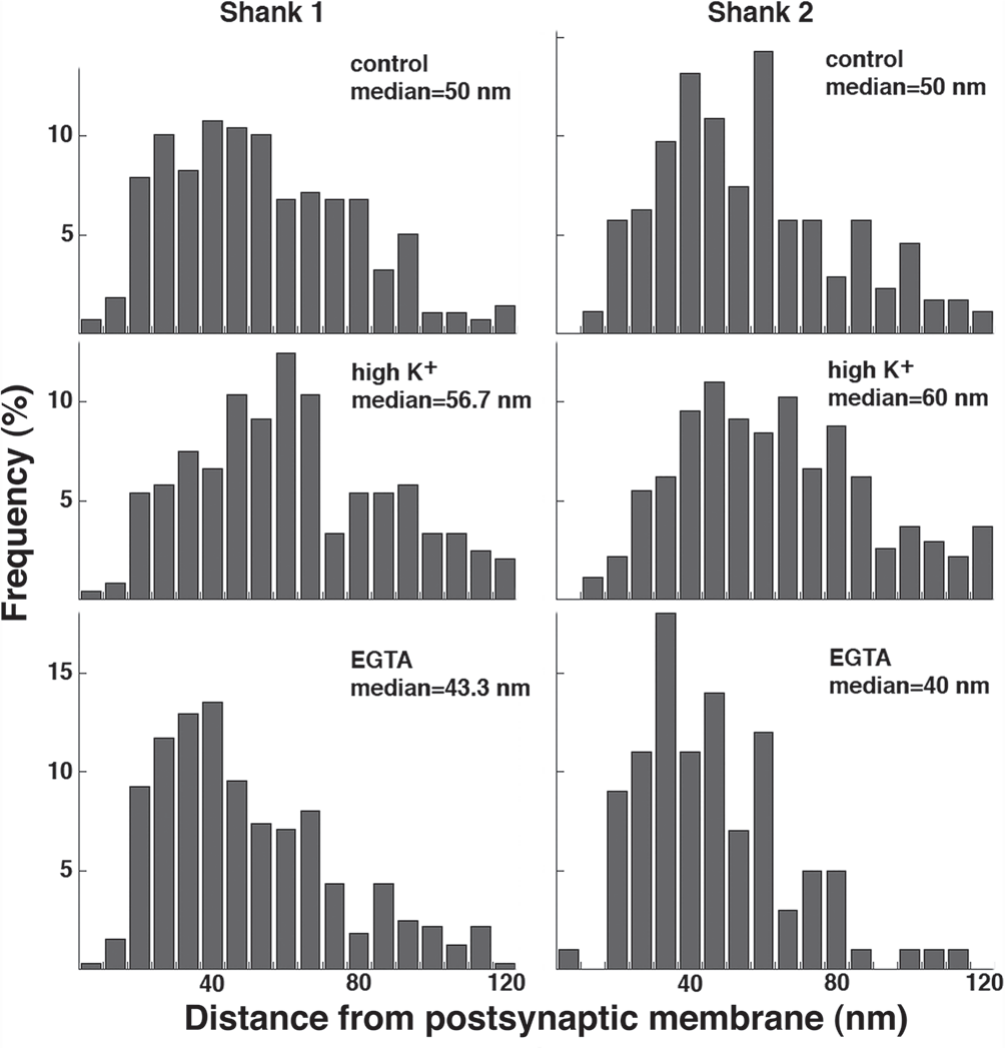
Histograms of distance measurements of labels for Shank 1 and Shank 2 under different experimental conditions. Sister cultures were labeled with antibodies against either Shank 1 (left) or Shank 2 (right) after identical experimental manipulations and processed in parallel. For each Shank isoform, the differences between control and high K^+^ or control and EGTA are statistically significant (Wilcoxon ran-sum test; same data set as exp 3 in Table 4).

Under basal conditions, label for Shank1 and Shank 2 are both located in a wider area in the PSD complex (Fig. 8, top), compared to that of GKAP (cf. Fig. 4D). These results indicate that the two isoforms are represented in both proximal and distal pools, and are not differentially located in the two pools in the PSD complex.

**Table 4:** Median distances of label for Shank 1 and Shank 2 in the PSD complex under different conditions.

		Median distance (nm) from postsynaptic membrane (n = number of labels measured)		
		Control	High K^+^	EGTA
**Exp 1**	**Shank 1**	46.7 (120)	50.0 (245) N. S.	
	**Shank 2**	50.0 (273)	60.0 (342) P<0.0001	
**Exp 2**	**Shank 1**	40.0 (243)	53.3 (320) P<0.0001	
	**Shank 2**	43.3 (244)	53.3 (193) P<0.0001	
**Exp 3**	**Shank 1**	50.0 (279)	56.7 (241) P<0.001	43.3 (325) P<0.05
	**Shank 2**	50.0 (355)	60.0 (273) P<0.005	40.0 (289) P<0.0001
**Exp 4**	**Shank 1**	46.7 (134)		40.0 (141) P<0.05
	**Shank 2**	53.3 (164)		36.7 (171) P<0.0001

Within each experiment, sister cultures were labeled with either Shank 1 or Shank 2 antibody and processed in parallel.

Statistical analysis: Wilcoxon rank-sum test. N. S.: not significant.

As reported previously, both Shank 1 and Shank 2 are recruited from nearby cytoplasm to the PSD complex upon depolarization with high K^+^ [12] albeit in different ratios. Here, comparing the distance histograms of the label for the two isoforms, median distances for both Shank 1 and Shank 2 increased upon high K^+^ treatment, and decreased after EGTA treatment (Fig. 8; Table 4). Our data in general indicate that the distal pools of both isoforms are preferentially affected after high K^+^ or EGTA treatment.

### Similar patterns of labeling for Shank and GKAP in a subpopulation of PSDs following treatment with EGTA

**Fig 9:**
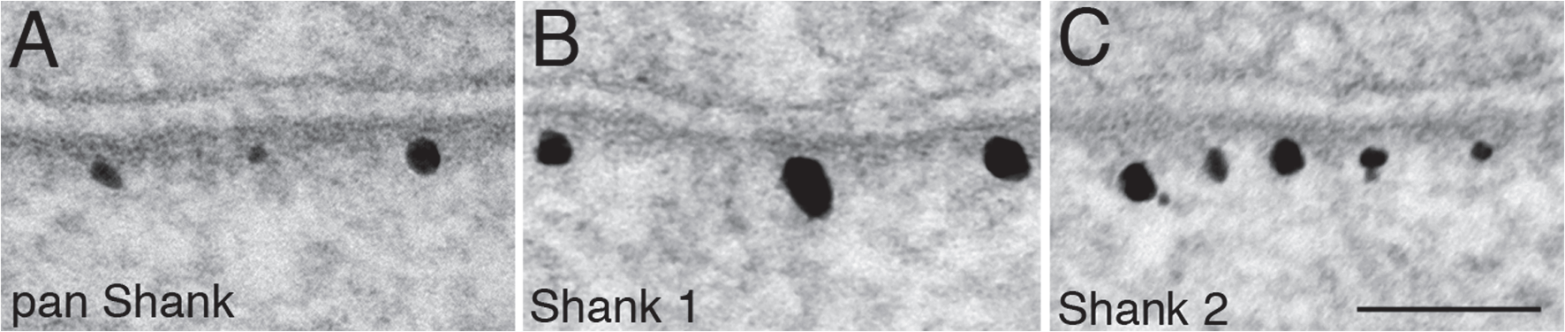
Under low calcium conditions, a subpopulation of synapses show an atypical labeling pattern for Shank resembling that for GKAP. In EGTA-treated samples, approximately one in six synapses showed an atypical labeling pattern for Shank, showing a narrow band close to the postsynaptic membrane. This pattern was seen with the three antibodies used here: pan Shank (A), Shank 1 (B), and Shank 2 (C), upon application of EGTA either in the presence (B) or absence of high K^+^ (A and C). Scale bar = 0.1 μm.

An interesting subpopulation of synapses emerged after EGTA treatment in which the label for Shank appeared in a narrow band close to the postsynaptic membrane (Fig. 9), a pattern resembling that for GKAP (cf. Fig. 4C). This atypical labeling pattern for Shank was apparent with pan Shank (Fig. 9A), Shank 1 (Fig. 9B) and Shank 2 (Fig. 9C) antibodies.

The frequency of this atypical labeling pattern for Shank is linked to low calcium conditions. In experiments using pan Shank antibody, when EGTA is included in calcium-free control medium, 17.2 ± 5.4% (three experiments) of synaptic profiles showed this pattern. In contrast, none of the synapses after high K^+^ treatment (four experiments) and only 3.5 ± 0.7% (four experiments) of synapses under control conditions showed this pattern. Thus, in these synapses under low calcium conditions, Shank molecules were absent in the distal area of the PSD complex but persisted in the proximal area near GKAP.

## Discussion

In the present study, immunogold-electron microscopy is used to examine the distributions of Shank and GKAP using antibodies raised to epitopes encompassing their mutual binding domains. An obvious caveat of such an approach is the possibility that binding of Shank and GKAP to each other may interfere with the binding of antibodies. However, the use of antibodies produced against peptides that encompass the binding domains as well as relatively long flanking sequences would minimize this possibility. Indeed, relatively robust and overlapping labeling for Shank and GKAP is observed at the proximal area of the PSD complex where the two groups of molecules are presumably associated.

It is reasonable to assume that if Shank and GKAP molecules were all bound to each other, immunogold labeling with antibodies recognizing epitopes around their mutual binding sites should have similar distributions within the PSD. The distribution of label for Shank is, however, broader than that of GKAP. In fact ~40% of Shank label lies within an area 60–120 nm from the postsynaptic membrane that contains virtually no GKAP label. This disparity in the distribution of label suggests the existence of two pools of Shank molecules at the PSD, a more proximal pool that can associate with GKAP and a more distal pool that is located too far away from GKAP to associate with it.

Depolarization with high K^+^ or NMDA treatment promotes recruitment of additional Shank to the PSD as well as an increase in the median distance of Shank label from the postsynaptic membrane (present data, [17]). Conversely, lowering Ca^2+^ levels preferentially removes distal Shank label from the PSD as indicated by a decrease in the median distance from the postsynaptic membrane. Preferential addition and removal of Shank from the distal region of the PSD in response to pharmacological manipulations supports the existence of two distinct pools of Shank at the PSD: a relatively stable proximal pool and a dynamic distal pool.

The distribution of GKAP at the PSD does not change under the experimental conditions applied in the present study. We speculate that the more stable proximal pool of Shank is associated with the stable layer of GKAP under these conditions. Indeed, application of EGTA reveals a group of synapses where the distribution of Shank is nearly identical to that of GKAP. In this group of synapses, the distal pool of Shank may have been depleted under the low Ca^2+^ conditions, leaving only the GKAP-bound proximal pool of Shank at the PSD.

Long-term (24–48 hr) changes in neuronal activity promote bi-directional changes in the levels of synapse-associated GKAP [18, 19]. However, our EM observations indicate that GKAP levels at the PSD remain stable upon 2 min depolarization with high K^+^. Previous studies by fluorescent microscopy reached a different conclusion on the short-term stability of GKAP in the spine. Acute bicuculline/4 AP treatment (1–15 min), which induces robust synaptic activity, increased the intensity of synaptic GKAP puncta [20, 21]. This discrepancy with our finding regarding short-term changes in GKAP labeling at the synapses may be due to differences in activation protocols. Alternatively, the increase in GKAP levels in spines observed by fluorescence microscopy may not reflect an increase in GKAP at the PSD.

There are three main isoforms of Shank, Shank 1, Shank 2 and Shank 3, encoded by three different genes. All three Shank isoforms have PDZ domains that enable association with GKAP as well as SAM domains that enable association between Shanks. Based on mutational studies, Sala et al. [14] concluded that synaptic localization of Shank 1 requires its PDZ domain. Using a similar approach, Boeckers et al. [15] concluded that the synaptic localization of Shank 2 and Shank 3 depends on their C-terminal SAM domains. These observations led us to test the possibility that the proximal and distal pools of Shank may correspond to the PDZ-dependent (Shank 1) and SAM-domain dependent subtypes (Shank 2 and 3) respectively. In this set of experiments the distributions of Shank 1 and 2, two Shank isoforms with the most suitable antibodies for immunogold labeling, were compared. Specific antibodies for Shank 1 (PDZ domain dependent subtype) and Shank 2 (SAM domain dependent subtype) produce similarly broad label distributions within the PSD, indicating proximal as well as distal localization of both subtypes. Moreover, both subtypes redistribute in a similar manner upon experimental manipulations.

The consensus on the molecular organization of the PSD is that it is a layered scaffold [1–4]. It is generally accepted that PSD-95 and other MAGUKs lay adjacent to the postsynaptic membrane and bind to GKAPs, GKAPs bind to Shanks, and Shanks, in turn, bind to Homers. Our observations, however, indicate that a pool of Shank molecules in the distal part of the PSD is not bound to GKAP, raising the question of how these distal Shank molecules are tethered to the PSD.

One possible explanation is Shank’s association with Homer. Purified Shank and Homer polymerize to make a mesh-like structure [22]. Homer molecules form tetramers through tail-to-tail interaction of two dimers, with the Shank-binding N-terminals of the individual Homer proteins at the two ends of the tetramer. Thus, a Homer tetramer can crosslink Shanks. Alternatively, distal Shanks may be tethered to GKAP-associated Shanks through Shank-Shank association via their C-terminal SAM domains [23]. Both scenarios are consistent with the high degree of overlap between the distributions of Shank and Homer labels [24] deeper within the PSD complex.

Interestingly, low calcium conditions preferentially deplete Shank in the distal area of the PSD complex but did not affect Homer labeling intensity [24], indicating that Shank and Homer are differentially affected by calcium at the PSD.

Shanks are scaffolds with multiple protein-protein interaction domains. In addition to association with other PSD scaffold proteins, GKAPs and Homers, Shanks can bind and organize other proteins, including Densin-180, dynamin-2, and actin regulating elements [1, 3]. The proximal and distal populations of Shank appear to organize two layers of the PSD complex with different responses to immediate activity. A membrane-associated protein such as Densin-180 [25] that is likely to be anchored to Shank at the proximal region would remain stable during activity, while recruitment of other Shank binding proteins such as IRSp53, Abp1 and cortactin to the distal layer could mediate acute regulation of the actin cytoskeleton in response to synaptic activity.
